# Prevalence of Mental Health Problems and Its Associated Factors Among Recovered COVID-19 Patients During the Pandemic: A Single-Center Study

**DOI:** 10.3389/fpsyt.2021.602244

**Published:** 2021-03-31

**Authors:** Mojgan Khademi, Roya Vaziri-Harami, Jamal Shams

**Affiliations:** ^1^Department of Psychiatry, School of Medicine, Imam Hossein Hospital Clinical Research Development Unit, Shahid Beheshti University of Medical Sciences, Tehran, Iran; ^2^Behavioral Research Center, Imam Hossein Hospital, Shahid Beheshti University of Medical Sciences, Tehran, Iran

**Keywords:** COVID-19, anxiety, depression, post-traumatic stress disorder, mental health

## Abstract

**Introduction:** The coronavirus disease 2019 (COVID-19), is profoundly affecting the mental health status. Although the burden of mental health problems has been reported in the general population and health care workers, little is known about the prevalence of mental health disorders among recovered COVID-19 patients and their associated factors.

**Methods:** A cross-sectional telephonic-study of recovered COVID-19 patients with and without a history of hospitalization was conducted from April 20 to June 20, 2020, in Tehran, Iran. We assessed the anxiety symptoms, depression, and post-traumatic stress disorder (PTSD) among participants, using the Patient Health Questionnaire (PHQ-4) and PTSD checklist for DSM-5 (PCL-5). Logistic regression analyses were used to explore the risk factors associated with mental health problems.

**Results:** A total of 602 individuals with a mean age of 53.2 years (SD: 14.7), completed the study. The rates of mental health symptoms among the respondents were 5.8% (95% CI: 4.2–7.8%) for anxiety, 5.0% (95% CI: 3.5–7.0%) for depression, and 3.8% (95% CI: 2.3–5.3%) for PTSD disorders. Moreover, being younger than 50 years and female gender was significantly associated with a higher probability of reporting anxiety (*p* < 0.01), and depression (*p* < 0.001 for being younger than 50 years, *p* < 0.02 for female gender).

**Conclusions:** The current study indicated that patients with COVID-19 presented features of anxiety, depression, and PTSD. These results may help implement appropriate mental health intervention policies for those at risk and minimize the mental health consequences of the COVID-19.

## Introduction

The coronavirus disease 2019 (COVID-19) outbreak caused by severe acute respiratory syndrome coronavirus 2 (SARS-CoV-2) has been rapidly transmitted in late January 2020 and aroused enormous attention globally. To date (August 29th, 2020), over 24 million confirmed cases and 830,000 deaths attributable to this disease had been reported. In response to this global health crisis, strict public health measures have been implemented to restrict the spread of the virus (1–5). As a result of the rapidly increasing numbers of infected cases, many patients along with the symptoms of the disease itself, have been experiencing psychological problems including depression, anxiety, and stress (6–11). The neuroinvasive potential of SARS-CoV-2 may affect brain function and mental health (12–17). Furthermore, treatment for COVID-19 may have adverse effects on mental health and contribute to anxiety (18).

According to a recent study, on the psychological assessment of 89 patients with COVID-19, 35% had mild symptoms, and 13% had moderate to severe symptoms (19). Likewise, in another study on hospital discharged patients with COVID-19, 10.4% were recognized as having mild to severe symptoms of anxiety and 19% were diagnosed with clinically significant symptoms of depression (20). Additionally, post-traumatic stress disorder (PTSD) due to COVID-19 was diagnosed in 12.4% of patients (20). Therefore, in response to the COVID-19 epidemic, psychological problems are an important issue. To address the mental health need of the patients with COVID-19, multiple measures such as telemedicine guidelines, mental health helpline, and strengthened mental health program has been implemented by several countries in the world (21–24). Recently-published studies on the psychological impact of COVID-19 are mainly focused on the general population and healthcare workers, who were worried about the risks of infection, resulting in psychological distress (25–29). To date, no studies have reported the prevalence of depression, anxiety, and PTSD symptoms for the recovered COVID-19 patients in Iran. Furthermore, a reliable estimate of the extent of mental disorders is needed to manage such patients. The present study was aimed to determine the prevalence of depression, anxiety, and PTSD symptoms and its associated factors in COVID-19 participants with and without a history of hospitalization in Iran.

## Methods

### Study Design and Participants

This study used a cross-sectional telephonic-study method conducted from April 20, to June 20, 2020, in a COVID-19 referral hospital in Tehran, Iran. Study participants included those with a severe form of COVID-19 who were hospitalized in the last month and individuals with mild to moderate form of COVID-19 who had been discharged from hospitals without hospitalization. Thus, our recovered patients were divided into two groups based on a history of hospitalization. Patients were contacted consecutively through telephone one and a half months after diagnosing the disease, by trained research psychiatric residents. Before filling out several questionnaires, all participants agreed with the consent forms over the phone call. Missed patients were contacted after 1 day to ensure their participation. The study protocol was approved by the research ethics committee of Shahid Beheshti University of Medical Sciences (IR.SBMU.RETECH.REC.1399.352).

### Screening Measures

Participants were asked to report their age, gender, and psychiatric history. An interview approved by the Ministry of Health of Iran conducted by mental health experts in disasters, including earthquakes, was used to assess the presence or absence of any psychological distress, functional impairment, and severe mental preoccupation (30). However, in our questions, the connection of these signs with COVID-19 was considered and asked. Based on these questions, if a person answers yes to even one question, he/she will be a candidate to participate in psychosocial interventions to prevent mental health disorders.

### Patient Health Questionnaire (PHQ-4)

To investigate the psychological symptoms, we used the Patient Health Questionnaire for depression and anxiety (the PHQ-4) (31). The PHQ-4 uses a two-item measure (the PHQ−2), consisting of core criteria for depression, as well as a two-item measure for anxiety (the GAD−2). Individuals with the PHQ-4 scores of 3 or above were classified as having anxiety and/or depression symptoms (31). Suicidal ideation at the right time of the interview-after completing the PHQ was asked. The following questions were asked: Ever since you fell ill, do you think you have no desire to live and would rather die? Have you ever thought of hurting yourself to die—or think of suicide—during the pandemic?

### The PTSD Checklist (PCL)

PTSD symptoms were assessed using the PTSD Checklist PCL-5 (DSM-5) (32). Anyone whose PCL-5 reached the cutoff score was subjected to a clinical interview based on the DSM-5 criteria to make a definitive diagnosis (33).

The PCL-5 is a self-report measure, consisting of 20 items that each item reflected the severity of a particular symptom, rated on a five-point Likert scale from 0 (not at all) to 4 (extremely) during the previous month. This questionnaire is not specific to a specific term and can be used in all disasters. It also has good validity and reliance in Iran (34).

### Statistical Analyses

The prevalence of symptoms of anxiety, depression, and PTSD, as well as psychological distress, functional impairment, severe mental preoccupation, and suicidal ideation, was calculated and reported as the percentages of cases in the different populations of the study. The 95% CIs were produced by the exact binomial methods. Chi-squared tests were used to compare the prevalence of mental health disorders in different populations. To explore factors (Independent variables: age, gender, and history of psychiatric disorders) potentially associated with dependent variables (anxiety, depression, PTSD, and suicidal ideation), logistic regression analyses were performed, and odds ratios (ORs) and 95% CIs were presented. *P* < 0.05 were considered statistically significant. All of the statistical analyses were performed using Statistical Package for Social Sciences (SPSS) statistical software version 22 (IBM Corp).

## Results

In total, 645 participants initially participated in the study; after excluding 43 individuals with missing/incomplete responses and without the consent forms, data on 602 patients remained for analysis (93.3%). Of the total sample, 386 participants (64.0%) were male, and the mean age was 53.2 years (Min: 15, Max: 93, SD: 14.7); 114 participants (19.0%) were aged 18–40 years.

### Prevalence of Severe Psychological Distress, Functional Impairment, Severe Mental Preoccupation, and Suicidal Ideation in Recovered COVID-19 Patients

The prevalence of the four mental health problems among the total participants was 10.0% (N: 61, 95% CI, 7.8–12.6%) for severe psychological distress, 4.5% (N: 27, 95% CI, 2.8–6.3%) for functional impairment, 7.0% (N: 42, 95% CI, 5.0–9.0%) for severe mental preoccupation, and 0.8% (N: 5, 95% CI, 0.2–1.7%) for suicidal ideation. The prevalence of these mental problems was high among respondents with a history of hospitalization, however, we did not find any significant difference among the patient groups (*p* > 0.05) (Table 1). Having a history of psychiatric disorders was reported in 2.8% of the respondents.

**Table 1:** Sociodemographic characteristics of the participants.

**Characteristic**	**All (No. 602)**	**Participants without a history of hospitalization No. (%)**	**Participants with a history of hospitalization No. (%)**	***P*-value**
**Gender**				
Male	386 (64.0)	119 (30.8)	267 (69.2)	0.85
Female	216 (36.0)	65 (30.1)	151 (69.9)	
**Age**				
Mean	53.2	55.1	48.7	0.00
**Psychological suffering**				
Yes	61 (10.0)	15 (24.6)	46 (75.4)	0.28
No	541 (90.0)	169 (31.2)	372 (68.8)	
**Dysfunction**				
Yes	27 (4.5)	4 (14.8)	23 (85.2)	0.06
No	575 (95.5)	180 (31.3)	395 (68.7)	
**Severe mental preoccupation**				
Yes	42 (7.0)	8 (19.0)	34 (81.0)	0.09
No	560 (93.0)	176 (31.4)	384 (68.6)	
**Suicidal ideation**				
Yes	5 (0.8)	2 (40.0)	3 (60.0)	0.64
No	597 (99.2)	182 (30.5)	415 (69.5)	

### Prevalence of Anxiety, Depression, and PTSD in Recovered COVID-19 Patients

The prevalence of anxiety, depression, and PTSD is shown in Table 2.

**Table 2:** Characteristics of COVID-19 recovered patients anxiety, depression, and PTSD.

**Characteristic**	**All (No. 602)**	**Participants without a history of hospitalization No. (%)**	**Participants with a history of hospitalization No. (%)**	***P*-value**
**Anxiety**				
No	567 (94.2)	176 (31.0)	391 (69.0)	0.30
Yes	35 (5.8)	8 (22.9)	27 (77.1)	
**Depression**				
No	571 (95.0)	176 (30.8)	395 (69.2)	0.55
Yes	31 (5.0)	8 (25.8)	23 (74.2)	
**PTSD**				
No	579 (96.2)	175 (31.0)	402 (69.4)	0.90
Yes	23 (3.8)	9 (24.3)	16 (69.6)	

For the prevalence of mental-relevant symptoms, 5.8% (N: 35, 95% CI: 4.2–7.8%) of the individuals completing the study presented clinically relevant anxiety symptoms, 5.0% (N: 31, 95% CI: 3.5–7.0%) depression symptoms, and 3.8% (N: 23, 95% CI: 2.3–5.3%) of the subjects were considered to have PTSD. These mental health outcomes were commonly found in patients with a history of hospitalization; however, there was no statistically significant difference in each of the patient groups' problems (*p* > 0.05). In participants with anxiety and depression symptoms, 40.0 and 42.0% were found to have a functional impairment, respectively (*p* = 0.01).

### Risk Factors of Mental Health Outcomes

As shown in Table 3, being younger than 50 years and female gender was significantly associated with a higher probability of reporting anxiety (*p* < 0.01), and depression (*p* < 0.001 for being younger than 50 years, *p* < 0.02 for female gender). Age younger than 50 years old also appeared to be protective for PTSD (Table 3).

**Table 3:** Regression analysis of risk factors for symptoms of anxiety, depression, PTSD, and suicidal ideation.

**Variables**	**Anxiety**		**Depression**		**PTSD**		**Suicidal ideation**	
	**OR**	***P*-value**	**OR**	***P-*value**	**OR**	***P*-value**	**OR**	***P*-value**
	**(95% CI)**		**(95% CI)**		**(95% CI)**		**(95% CI)**	
**Gender**								
Male	[Reference]	0.01	[Reference]	0.02	[Reference]	0.06	[Reference]	0.07
Female	2.6 (1.2–5.4)		2.2 (1.1–4.4)		3.0 (0.9–9.0)		7.2 (0.8–15.4)	
**Age (years)**								
≥50	[Reference]	0.01	[Reference]	0.00	[Reference]	0.04	[Reference]	0.2
15–49	3.7 (1.7–8.0)		3.5 (1.7–7.0)		0.3 (0.1–0.9)		3.2 (0.5–9.5)	
**History of Psychiatric Disorders**								
No	[Reference]		[Reference]		[Reference]		[Reference]	0.06
Yes	1.1(0.2–9.0)	0.8	1.0 (0.1–7.8)	0.9	3.0 (0.3–12.0)	0.9	9.2 (0.8–18.0)	

## Discussion

The present study showed the prevalence rates of anxiety, depression, and PTSD symptoms among Iranian COVID-19 recovered patients with and without a history of hospitalization during the pandemic. These findings provide a comprehensive profile of psychological status in the population with a history of COVID-19 in Iran and contribute to developing specific mental health management and intervention policies.

The results revealed that 10% of the respondents experienced severe psychological distress, 4.5% had a functional impairment, 7% experienced severe mental preoccupation, and 0.8% reported suicidal ideation linked to the COVID-19. Studies on the Italian population underlined a 41% prevalence of psychological distress due to the COVID-19 quarantine (35). Lee et al., have also indicated that the rate of functional impairment was 35.0%, among adults with dysfunctional coronavirus anxiety (36). They have also shown that individuals who were functionally impaired by their anxiety of the coronavirus exhibited more significant suicidal ideation, than those who were anxious, but not impaired by the disease (37). The different participants in the studies and various questionnaires to assess these psychological dimensions made a statistical comparison impossible. However, it is possible to observe that our results showed a worse psychological condition among the recovered patients with COVID-19.

The prevalence of anxiety and depression symptoms in the present study was lower than those in a similar study that was conducted among the discharged COVID-19 patients in China, which indicated that more than 10% of the participant manifested moderate to severe symptoms of anxiety and depression (20). Another study that was aimed to explore the prevalence and factors linked to anxiety and depression in hospitalized patients with COVID-19 found that 34.7 and 28.4% of patients had symptoms of anxiety and depression, respectively (38).

The rate of these psychiatric symptoms and disorders in our study was compared with reports before the pandemic, during which rates of depression and anxiety in the population aged 18 years and over living in Iran were ~4.2 and 8.3%, respectively (39).

It is noteworthy that 3.8% of COVID-19 recovered patients in the current study suffered from PTSD, and these symptoms may lead to adverse outcomes, such as impaired functional performance and lower quality of life (40). Our findings were lower than that of Bo et al., in which the prevalence of significant PTSD symptoms associated with the COVID-19 was 96.2% in China (40). The remarkable differences between these studies could be attributed to different clinical diagnoses (e.g., discharged patients vs. clinically stable COVID-19 inpatients from quarantine facilities) and other measurements (e.g., self-reported questionnaires on PTSD vs. clinical diagnosis of PTSD established by psychiatrists in the current study).

The rapid transmission of the disease and social discrimination toward COVID-19 patients may result in a higher prevalence of psychiatric disorders associated with the COVID-19. The adverse effect of COVID-19 on mental health may not end with discharge from the hospital, and many individuals continue to report moderate to severe forms of the disorders.

In the current study, the prevalence of anxiety, depression, and PTSD in COVID-19 recovered patients was comparable to those in the general population (39). The adverse mental health consequences of COVID-19 are more likely to happen in the recovered patients due to the fear of progression of their illness, disability, or premature death. Recent studies have also confirmed that the recovered patients with COVID-19 presented more features of anxiety and depression than the general public (28, 38). The higher risk of mental health disorders among the recovered patients with COVID-19 may also be attributable to the adverse effects of medications that are used to treat the disease.

The present study also found that people younger than 50 years, and female gender, reported greater symptoms of depression, and anxiety. Likewise, Shi et al., and Kong et al., indicated that a high probability of symptoms of depression and anxiety was found among people who had younger age, females patients, and those having a personal history of psychiatric disorders (28, 38). Furthermore, Casagrande et al., showed that youth and women have a greater risk of developing COVID-19-related distress (35). These findings suggest that such people, particularly those with severe illness, should receive mental health support after hospital discharge. Accordingly, studies showed that mental support is one of the key factors linked to anxiety and depression for patients with COVID-19, and less psychological support was associated with more anxious and depression symptoms (38, 41). Several psychological interventions have been developed to respond to psychological pressures. Building a tele-mental health services team is one of the psychological interventions that could play an important role in monitoring psychosocial needs and delivering psychosocial support to patients with COVID-19 (42). This intervention can effectively decline the mental illness outcome and restricted mobility while reducing patient and clinician infection risk. Studies from Italy, Australia, and China also confirmed that tele-mental consultation could provide satisfactory results and has high acceptance among patients with mental illness (22, 43, 44). Furthermore, education and training regarding psychosocial concerns should be provided to health system authorities, and health care professionals to identify, develop, and disseminate resources related to disaster mental health (45).

The results of the current study need to be considered in the context of some limitations. First, it was a cross-sectional telephonic-study, thus limiting causal interpretations. Second, data on previous mental disorders before COVID-19 were not fully assessed, thus it remains unknown whether these symptoms are associated with the COVID-19 pandemic or pre-existed. Third, the severe symptoms of COVID-19 were not assessed. Fourth, our study was single-centered, which limits the generalizability of the findings. Fifth, as the PCL-5, was a self-report instrument, and due to the specific circumstances of the pandemic, participants completed it in a phone interview; the same sentences of this questionnaire were read by the interviewer to the participant and their answers were written down. Finally, we did not include the follow-up data because another study being conducted by the authors. Future studies are needed to determine the possible long-term mental health outcomes associated with the COVID-19.

In conclusion, our study indicated that patients with COVID-19 presented anxiety, depression, and PTSD symptoms. These results may help implement appropriate mental health intervention policies for those at risk and minimize the mental health consequences of the COVID-19.

## Data Availability Statement

The raw data supporting the conclusions of this article will be made available by the authors, without undue reservation.

## Ethics Statement

The studies involving human participants were reviewed and approved by Shahid Beheshti University of Medical Sciences. The patients/participants provided their written informed consent to participate in this study.

## Author Contributions

All authors listed have made a substantial, direct and intellectual contribution to the work, and approved it for publication.

## Conflict of Interest

The authors declare that the research was conducted in the absence of any commercial or financial relationships that could be construed as a potential conflict of interest.
